# Dichlorido{2-[3-(dimethyl­ammonio)­propyl­imino­meth­yl]phenolato}zinc(II) hemihydrate

**DOI:** 10.1107/S1600536808033977

**Published:** 2008-10-25

**Authors:** Xue-Wen Zhu

**Affiliations:** a Key Laboratory of Surface and Interface Science of Henan, School of Materials and Chemical Engineering, Zhengzhou University of Light Industry, Zhengzhou 450002, People’s Republic of China

## Abstract

The title complex, [ZnCl_2_(C_12_H_18_N_2_O)]·0.5H_2_O, is a mononuclear zinc(II) compound derived from the zwitterionic form of the Schiff base 2-[3-(dimethyl­amino)propyl­imino­meth­yl]­phenol. The Zn^II^ atom is four-coordinated by the imine N and the phenolate O atoms of the Schiff base ligand, and by two chloride ions, in a distorted tetra­hedral coordination geometry. The dimethyl­ammonio group is disordered over two positions with site occupancies of 0.51 (3) and 0.49 (3). In the asymmetric unit, there is also a disordered water mol­ecule with a partial occupancy of 0.5. In the crystal structure, the water mol­ecules are linked to the Schiff base complex mol­ecules through inter­molecular N—H⋯O hydrogen bonds. Mol­ecules are further linked through additional inter­molecular N—H⋯O hydrogen bonds, forming chains running along the *b* axis.

## Related literature

For a general background on the chemistry of Schiff base complexes, see: Ali *et al.* (2008 ▶); Biswas *et al.* (2008 ▶); Chen *et al.* (2008 ▶); Darensbourg & Frantz (2007 ▶); Habibi *et al.* (2007 ▶); Kawamoto *et al.* (2008 ▶); Lipscomb & Sträter (1996 ▶); Tomat *et al.* (2007 ▶); Wu *et al.* (2008 ▶); Yuan *et al.* (2007 ▶). For related structures, see: Zhu & Yang (2008*a* ▶,*b* ▶,*c* ▶,*d* ▶); Qiu (2006*a* ▶,*b* ▶); Wei *et al.* (2007 ▶); Zhu *et al.* (2007 ▶).
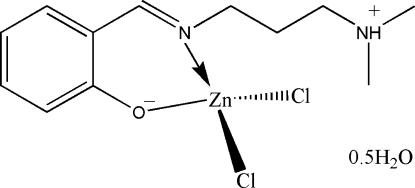


## Experimental

### 

#### Crystal data

[ZnCl_2_(C_12_H_18_N_2_O)]·0.5H_2_O
*M* *_r_* = 351.58
Orthorhombic, 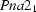

*a* = 13.335 (2) Å
*b* = 16.384 (2) Å
*c* = 7.212 (1) Å
*V* = 1575.7 (4) Å^3^
*Z* = 4
Mo *K*α radiation
μ = 1.89 mm^−1^
*T* = 298 (2) K
0.23 × 0.23 × 0.22 mm

#### Data collection

Bruker APEXII CCD area-detector diffractometer
Absorption correction: multi-scan (*SADABS*; Sheldrick, 2004 ▶) *T*_min_ = 0.650, *T*_max_ = 0.661
12635 measured reflections
3426 independent reflections
2915 reflections with *I* > 2σ(*I*)
*R*_int_ = 0.035

#### Refinement

*R*[*F* ^2^ > 2σ(*F* ^2^)] = 0.041
*wR*(*F* ^2^) = 0.103
*S* = 1.08
3426 reflections
206 parameters
4 restraints
H atoms treated by a mixture of independent and constrained refinement
Δρ_max_ = 0.42 e Å^−3^
Δρ_min_ = −0.40 e Å^−3^
Absolute structure: Flack (1983 ▶), 1569 Friedel pairs
Flack parameter: 0.03 (2)

### 

Data collection: *APEX2* (Bruker, 2004 ▶); cell refinement: *SAINT* (Bruker, 2004 ▶); data reduction: *SAINT*; program(s) used to solve structure: *SHELXS97* (Sheldrick, 2008 ▶); program(s) used to refine structure: *SHELXL97* (Sheldrick, 2008 ▶); molecular graphics: *SHELXTL* (Sheldrick, 2008 ▶); software used to prepare material for publication: *SHELXTL*.

## Supplementary Material

Crystal structure: contains datablocks global, I. DOI: 10.1107/S1600536808033977/rz2256sup1.cif

Structure factors: contains datablocks I. DOI: 10.1107/S1600536808033977/rz2256Isup2.hkl

Additional supplementary materials:  crystallographic information; 3D view; checkCIF report

## Figures and Tables

**Table 1:** Selected bond lengths (Å)

Zn1—O1	1.954 (3)
Zn1—N1	2.003 (4)
Zn1—Cl1	2.2182 (13)
Zn1—Cl2	2.2692 (18)

**Table 2:** Hydrogen-bond geometry (Å, °)

*D*—H⋯*A*	*D*—H	H⋯*A*	*D*⋯*A*	*D*—H⋯*A*
N2′—H2′*A*⋯O1^i^	0.91	1.88	2.762 (14)	164
N2—H2*C*⋯O1^i^	0.91	1.87	2.773 (12)	170

Symmetry code: (i) 
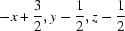
.

**Fig. 1.**
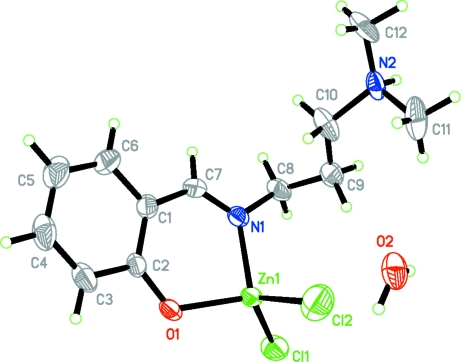
The molecular structure of the title compound with ellipsoids drawn at the 30% probability level. Only the major component of disorder is shown.

**Fig. 2.**
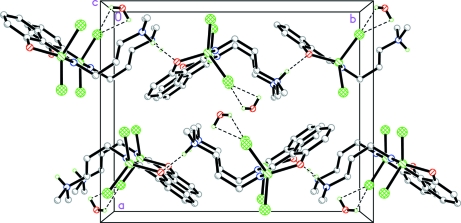
The crystal packing of the title compound viewed along the c axis. Hydrogen atoms not involved in hydrogen bonding interactions (dashed lines) are omitted for clarity. Only the major component of disorder is shown.

## supplementary crystallographic information

### Comment

Schiff bases have widely been used as versatile ligands in coordination chemistry (Biswas *et al.*, 2008; Wu *et al.*, 2008; Kawamoto *et al.*, 2008; Ali *et al.*, 2008; Habibi *et al.*, 2007), and their metal complexes are of great interest in many fields (Chen *et al.*, 2008; Yuan *et al.*, 2007; Tomat *et al.*, 2007; Darensbourg & Frantz, 2007). Zinc(II) is an important element in biological systems and functions as an active site of hydrolytic enzymes, such as carboxypeptidase and carbonic anhydrase  where it is in a hard-donor coordination environment of nitrogen and oxygen ligands (Lipscomb & Sträter, 1996). Recently, we have reported some Schiff base complexes (Zhu & Yang, 2008*a*,*b*,*c*,*d*). In this paper, the synthesis and structural characterization of a new zinc(II) complex (Fig. 1) of the Schiff base ligand 2-[(3-dimethylaminopropylimino)methyl]phenol is reported.

The zinc(II) atom in the title compound is four-coordinated by the imine N and phenolate O atoms of the zwitterionic form of the Schiff base ligand, and by two Cl^-^ ions in a tetrahedral coordination geometry. The coordinate bond lengths (Table 1) are typical and comparable to the corresponding values observed in other similar zinc(II) Schiff base complexes (Zhu *et al.*, 2007; Wei *et al.*, 2007; Qiu, 2006*a*,*b*).

In the crystal structure, the water molecules are linked to the Schiff base complex molecules through intermolecular N–H···O hydrogen bonds (Table 2). The molecules are further linked through intermolecular N–H···O hydrogen bonds (Table 2), forming chains running along the *b* axis (Fig. 2).

### Experimental

The Schiff base compound was prepared by the condensation of equimolar amounts of salicylaldehyde with *N*,*N*-dimethylpropane-1,3-diamine in a methanol solution. The complex was prepared by the following method: to an anhydrous methanol solution (5 ml) of ZnCl_2_ (13.7 mg, 0.1 mmol) was added a methanol solution (10 ml) of the Schiff base compound (20.6 mg, 0.1 mmol) with stirring. The mixture was stirred for 30 min at room temperature and filtered. Upon keeping the filtrate in air for a few days, colourless block-shaped crystals were formed at the bottom of the vessel on slow evaporation of the solvent.

### Refinement

H atoms bound to C and N atoms were placed in geometrically idealized positions and constrained to ride on their parent atoms, with C–H distances in the range 0.93–0.97 Å, N–H distances of 0.91 Å, and with *U*_iso_(H) = 1.2*U*_eq_(C,N) and 1.5*U*_eq_(methyl C). The dimethylammmonium group is disordered over two distinct sites, with occupancies of 0.51 (3) and 0.49 (3), respectively. The lattice water molecule is also disordered, with an occupancy restrained to 0.50. The water H atoms were placed at calculated positions and refined with the O—H and H···H lengths constrained to 0.85 (1) and 1.37 (2) Å, respectively, and with the isotropic thermal parameter fixed at 0.08 Å^2^.

### Crystal data

**Table:**

[ZnCl_2_(C_12_H_18_N_2_O)]·0.5H_2_O	*F*(000) = 724
*M**_r_* = 351.58	*D*_x_ = 1.482 Mg m^−^^3^
Orthorhombic, *P**n**a*2_1_	Mo *K*α radiation, λ = 0.71073 Å
Hall symbol: P 2c -2n	Cell parameters from 3872 reflections
*a* = 13.335 (2) Å	θ = 2.4–25.3°
*b* = 16.384 (2) Å	µ = 1.89 mm^−^^1^
*c* = 7.212 (1) Å	*T* = 298 K
*V* = 1575.7 (4) Å^3^	Block, colorless
*Z* = 4	0.23 × 0.23 × 0.22 mm

### Data collection

**Table:**

Bruker APEXII CCD area-detector diffractometer	3426 independent reflections
Radiation source: fine-focus sealed tube	2915 reflections with *I* > 2σ(*I*)
graphite	*R*_int_ = 0.035
ω scans	θ_max_ = 27.0°, θ_min_ = 2.0°
Absorption correction: multi-scan (SADABS; Sheldrick, 2004)	*h* = −17→17
*T*_min_ = 0.650, *T*_max_ = 0.661	*k* = −20→20
12635 measured reflections	*l* = −9→9

### Refinement

**Table:**

Refinement on *F*^2^	Secondary atom site location: difference Fourier map
Least-squares matrix: full	Hydrogen site location: inferred from neighbouring sites
*R*[*F*^2^ > 2σ(*F*^2^)] = 0.041	H atoms treated by a mixture of independent and constrained refinement
*wR*(*F*^2^) = 0.103	*w* = 1/[σ^2^(*F*_o_^2^) + (0.0381*P*)^2^ + 0.643*P*] where *P* = (*F*_o_^2^ + 2*F*_c_^2^)/3
*S* = 1.08	(Δ/σ)_max_ = 0.001
3426 reflections	Δρ_max_ = 0.42 e Å^−^^3^
206 parameters	Δρ_min_ = −0.40 e Å^−^^3^
4 restraints	Absolute structure: Flack (1983), 1569 Friedel pairs
Primary atom site location: structure-invariant direct methods	Flack parameter: 0.03 (2)

### Special details

**Table:**

Geometry. All esds (except the esd in the dihedral angle between two l.s. planes) are estimated using the full covariance matrix. The cell esds are taken into account individually in the estimation of esds in distances, angles and torsion angles; correlations between esds in cell parameters are only used when they are defined by crystal symmetry. An approximate (isotropic) treatment of cell esds is used for estimating esds involving l.s. planes.
Refinement. Refinement of F^2^ against ALL reflections. The weighted R-factor wR and goodness of fit S are based on F^2^, conventional R-factors R are based on F, with F set to zero for negative F^2^. The threshold expression of F^2^ > 2sigma(F^2^) is used only for calculating R-factors(gt) etc. and is not relevant to the choice of reflections for refinement. R-factors based on F^2^ are statistically about twice as large as those based on F, and R- factors based on ALL data will be even larger.

### Fractional atomic coordinates and isotropic or equivalent isotropic displacement parameters (Å^2^)

**Table:**

	*x*	*y*	*z*	*U*_iso_*/*U*_eq_	Occ. (<1)
Zn1	0.73458 (3)	1.10664 (2)	1.00222 (10)	0.04900 (15)	
Cl1	0.59091 (11)	1.09513 (7)	1.1550 (2)	0.0746 (4)	
Cl2	0.86270 (14)	1.03257 (11)	1.1229 (3)	0.1022 (5)	
N1	0.7159 (3)	1.0824 (2)	0.7323 (5)	0.0449 (7)	
O1	0.7749 (2)	1.21988 (16)	0.9610 (4)	0.0563 (8)	
O2	0.9856 (5)	0.9368 (4)	0.1435 (12)	0.0704 (18)	0.50
C1	0.8128 (3)	1.2011 (2)	0.6371 (6)	0.0488 (9)	
C2	0.8218 (3)	1.2425 (2)	0.8055 (6)	0.0464 (9)	
C3	0.8817 (4)	1.3118 (3)	0.8081 (9)	0.0656 (13)	
H3	0.8897	1.3401	0.9189	0.079*	
C4	0.9288 (4)	1.3393 (3)	0.6546 (10)	0.0783 (16)	
H4	0.9668	1.3868	0.6615	0.094*	
C5	0.9217 (4)	1.2989 (3)	0.4892 (11)	0.0835 (17)	
H5	0.9564	1.3172	0.3853	0.100*	
C6	0.8631 (4)	1.2317 (3)	0.4807 (8)	0.0736 (14)	
H6	0.8558	1.2049	0.3678	0.088*	
C7	0.7537 (3)	1.1280 (3)	0.6068 (6)	0.0506 (10)	
H7	0.7423	1.1129	0.4843	0.061*	
C8	0.6601 (3)	1.0080 (2)	0.6728 (7)	0.0575 (11)	
H8A	0.5968	1.0053	0.7389	0.069*	
H8B	0.6455	1.0117	0.5413	0.069*	
C9	0.7184 (4)	0.9327 (3)	0.7093 (7)	0.0674 (13)	
H9A	0.6748	0.8858	0.6947	0.081*	
H9B	0.7419	0.9335	0.8365	0.081*	
N2	0.8576 (13)	0.8452 (7)	0.543 (2)	0.055 (4)	0.51 (3)
H2C	0.8090	0.8071	0.5247	0.066*	0.51 (3)
C10	0.8059 (4)	0.9240 (3)	0.5831 (9)	0.0800 (18)	0.51 (3)
H10A	0.8570	0.9608	0.6296	0.096*	0.51 (3)
H10B	0.7849	0.9456	0.4641	0.096*	0.51 (3)
C11	0.9122 (17)	0.8214 (9)	0.713 (4)	0.091 (8)	0.51 (3)
H11A	0.8683	0.8257	0.8176	0.137*	0.51 (3)
H11B	0.9687	0.8569	0.7296	0.137*	0.51 (3)
H11C	0.9351	0.7661	0.7008	0.137*	0.51 (3)
C12	0.9244 (10)	0.8403 (9)	0.389 (3)	0.082 (6)	0.51 (3)
H12A	0.9523	0.7864	0.3816	0.123*	0.51 (3)
H12B	0.9775	0.8793	0.4036	0.123*	0.51 (3)
H12C	0.8883	0.8519	0.2765	0.123*	0.51 (3)
N2'	0.8443 (12)	0.8327 (9)	0.641 (4)	0.071 (5)	0.49 (3)
H2'A	0.7970	0.7970	0.5998	0.085*	0.49 (3)
C10'	0.8059 (4)	0.9240 (3)	0.5831 (9)	0.0800 (18)	0.49 (3)
H10C	0.8566	0.9650	0.6072	0.096*	0.49 (3)
H10D	0.7860	0.9265	0.4538	0.096*	0.49 (3)
C11'	0.8661 (16)	0.8106 (11)	0.824 (4)	0.101 (8)	0.49 (3)
H11D	0.8812	0.7534	0.8288	0.152*	0.49 (3)
H11E	0.8091	0.8220	0.9010	0.152*	0.49 (3)
H11F	0.9228	0.8413	0.8668	0.152*	0.49 (3)
C12'	0.9351 (17)	0.8164 (14)	0.534 (6)	0.139 (12)	0.49 (3)
H12D	0.9592	0.7626	0.5629	0.209*	0.49 (3)
H12E	0.9855	0.8559	0.5658	0.209*	0.49 (3)
H12F	0.9203	0.8199	0.4044	0.209*	0.49 (3)
H2A	0.984 (7)	0.973 (5)	0.228 (11)	0.080*	0.50
H2B	0.930 (4)	0.911 (5)	0.146 (15)	0.080*	0.50

### Atomic displacement parameters (Å^2^)

**Table:**

	*U*^11^	*U*^22^	*U*^33^	*U*^12^	*U*^13^	*U*^23^
Zn1	0.0640 (3)	0.0390 (2)	0.0440 (2)	−0.00734 (18)	0.0112 (3)	−0.0061 (3)
Cl1	0.0750 (8)	0.0720 (8)	0.0768 (8)	−0.0113 (6)	0.0324 (7)	−0.0094 (6)
Cl2	0.1140 (13)	0.1004 (11)	0.0921 (11)	−0.0145 (9)	−0.0136 (10)	0.0143 (9)
N1	0.0476 (18)	0.0393 (16)	0.0478 (18)	0.0030 (14)	0.0062 (15)	−0.0058 (14)
O1	0.0774 (18)	0.0361 (12)	0.055 (2)	−0.0102 (12)	0.0190 (14)	−0.0142 (12)
O2	0.056 (4)	0.054 (4)	0.102 (6)	0.011 (3)	0.005 (4)	0.015 (4)
C1	0.052 (2)	0.0417 (19)	0.053 (2)	0.0019 (17)	0.0052 (18)	0.0038 (18)
C2	0.051 (2)	0.0308 (17)	0.058 (2)	−0.0004 (16)	0.0125 (19)	0.0006 (16)
C3	0.067 (3)	0.042 (2)	0.088 (4)	−0.008 (2)	0.011 (3)	−0.014 (2)
C4	0.064 (3)	0.048 (2)	0.123 (5)	−0.009 (2)	0.031 (3)	0.009 (3)
C5	0.079 (3)	0.072 (3)	0.100 (4)	0.002 (2)	0.041 (4)	0.019 (4)
C6	0.088 (3)	0.070 (3)	0.063 (3)	0.001 (2)	0.030 (3)	0.010 (3)
C7	0.062 (3)	0.049 (2)	0.041 (2)	−0.0007 (18)	0.0022 (19)	−0.0092 (19)
C8	0.059 (3)	0.046 (2)	0.068 (3)	−0.0111 (19)	0.001 (2)	−0.018 (2)
C9	0.098 (4)	0.043 (2)	0.061 (3)	−0.021 (2)	−0.006 (3)	−0.010 (2)
N2	0.070 (7)	0.026 (3)	0.069 (9)	−0.004 (3)	−0.020 (7)	−0.001 (5)
C10	0.078 (3)	0.037 (2)	0.124 (5)	−0.002 (2)	−0.008 (3)	−0.020 (3)
C11	0.085 (14)	0.052 (7)	0.136 (19)	0.000 (7)	−0.051 (14)	0.024 (9)
C12	0.072 (8)	0.049 (7)	0.125 (14)	0.020 (5)	0.017 (9)	−0.028 (7)
N2'	0.067 (8)	0.044 (7)	0.101 (14)	0.004 (5)	−0.006 (9)	−0.029 (8)
C10'	0.078 (3)	0.037 (2)	0.124 (5)	−0.002 (2)	−0.008 (3)	−0.020 (3)
C11'	0.094 (12)	0.051 (8)	0.16 (2)	0.014 (8)	−0.054 (13)	−0.027 (10)
C12'	0.094 (12)	0.127 (16)	0.20 (4)	0.010 (12)	0.068 (19)	−0.002 (19)

### Geometric parameters (Å, °)

**Table:**

Zn1—O1	1.954 (3)	C9—C10	1.487 (8)
Zn1—N1	2.003 (4)	C9—H9A	0.9700
Zn1—Cl1	2.2182 (13)	C9—H9B	0.9700
Zn1—Cl2	2.2692 (18)	N2—C12	1.430 (19)
N1—C7	1.277 (6)	N2—C11	1.47 (2)
N1—C8	1.491 (5)	N2—C10	1.491 (15)
O1—C2	1.336 (5)	N2—H2C	0.9100
O2—H2A	0.852 (10)	C10—H10A	0.9700
O2—H2B	0.848 (10)	C10—H10B	0.9700
C1—C2	1.396 (6)	C11—H11A	0.9600
C1—C6	1.405 (6)	C11—H11B	0.9600
C1—C7	1.451 (6)	C11—H11C	0.9600
C2—C3	1.389 (6)	C12—H12A	0.9600
C3—C4	1.350 (8)	C12—H12B	0.9600
C3—H3	0.9300	C12—H12C	0.9600
C4—C5	1.368 (9)	N2'—C11'	1.40 (3)
C4—H4	0.9300	N2'—C12'	1.46 (2)
C5—C6	1.352 (7)	N2'—H2'A	0.9100
C5—H5	0.9300	C11'—H11D	0.9600
C6—H6	0.9300	C11'—H11E	0.9600
C7—H7	0.9300	C11'—H11F	0.9600
C8—C9	1.481 (7)	C12'—H12D	0.9600
C8—H8A	0.9700	C12'—H12E	0.9600
C8—H8B	0.9700	C12'—H12F	0.9600
			
O1—Zn1—N1	94.29 (13)	C10—C9—H9B	109.1
O1—Zn1—Cl1	113.19 (9)	H9A—C9—H9B	107.8
N1—Zn1—Cl1	111.04 (11)	C12—N2—C11	108.9 (14)
O1—Zn1—Cl2	111.03 (11)	C12—N2—C10	119.2 (9)
N1—Zn1—Cl2	111.10 (11)	C11—N2—C10	107.3 (12)
Cl1—Zn1—Cl2	114.48 (7)	C12—N2—H2C	106.9
C7—N1—C8	118.1 (4)	C11—N2—H2C	106.9
C7—N1—Zn1	121.6 (3)	C10—N2—H2C	106.9
C8—N1—Zn1	120.3 (3)	C9—C10—N2	124.4 (8)
C2—O1—Zn1	121.3 (2)	C9—C10—H10A	106.2
H2A—O2—H2B	108 (3)	N2—C10—H10A	106.2
C2—C1—C6	119.0 (4)	C9—C10—H10B	106.2
C2—C1—C7	125.4 (4)	N2—C10—H10B	106.2
C6—C1—C7	115.6 (4)	H10A—C10—H10B	106.4
O1—C2—C3	119.0 (4)	N2—C11—H11A	109.5
O1—C2—C1	123.7 (3)	N2—C11—H11B	109.5
C3—C2—C1	117.2 (4)	H11A—C11—H11B	109.5
C4—C3—C2	122.0 (5)	N2—C11—H11C	109.5
C4—C3—H3	119.0	H11A—C11—H11C	109.5
C2—C3—H3	119.0	H11B—C11—H11C	109.5
C3—C4—C5	121.4 (5)	N2—C12—H12A	109.5
C3—C4—H4	119.3	N2—C12—H12B	109.5
C5—C4—H4	119.3	H12A—C12—H12B	109.5
C6—C5—C4	118.3 (5)	N2—C12—H12C	109.5
C6—C5—H5	120.8	H12A—C12—H12C	109.5
C4—C5—H5	120.8	H12B—C12—H12C	109.5
C5—C6—C1	122.0 (6)	C11'—N2'—C12'	106.1 (19)
C5—C6—H6	119.0	C11'—N2'—H2'A	106.6
C1—C6—H6	119.0	C12'—N2'—H2'A	106.6
N1—C7—C1	126.2 (4)	N2'—C11'—H11D	109.5
N1—C7—H7	116.9	N2'—C11'—H11E	109.5
C1—C7—H7	116.9	H11D—C11'—H11E	109.5
C9—C8—N1	111.5 (4)	N2'—C11'—H11F	109.5
C9—C8—H8A	109.3	H11D—C11'—H11F	109.5
N1—C8—H8A	109.3	H11E—C11'—H11F	109.5
C9—C8—H8B	109.3	N2'—C12'—H12D	109.5
N1—C8—H8B	109.3	N2'—C12'—H12E	109.5
H8A—C8—H8B	108.0	H12D—C12'—H12E	109.5
C8—C9—C10	112.6 (4)	N2'—C12'—H12F	109.5
C8—C9—H9A	109.1	H12D—C12'—H12F	109.5
C10—C9—H9A	109.1	H12E—C12'—H12F	109.5
C8—C9—H9B	109.1		

### Hydrogen-bond geometry (Å, °)

**Table:**

*D*—H···*A*	*D*—H	H···*A*	*D*···*A*	*D*—H···*A*
N2'—H2'A···O1^i^	0.91	1.88	2.762 (14)	164
N2—H2C···O1^i^	0.91	1.87	2.773 (12)	170

Symmetry codes: (i) −*x*+3/2, *y*−1/2, *z*−1/2.

## References

[bb1] Ali, H. M., Mohamed Mustafa, M. I., Rizal, M. R. & Ng, S. W. (2008). *Acta Cryst.* E**64**, m718–m719. 10.1107/S1600536808011161 PMC2961161 21202245

[bb2] Biswas, C., Drew, M. G. B. & Ghosh, A. (2008). *Inorg. Chem.* **47**, 4513–4519. 10.1021/ic8002542 18459727

[bb3] Bruker (2004). *APEX2* and *SAINT* Bruker AXS Inc., Madison, Wisconsin, USA.

[bb4] Chen, Z., Morimoto, H., Matsunaga, S. & Shibasaki, M. (2008). *J. Am. Chem. Soc.* **130**, 2170–2171. 10.1021/ja710398q 18225906

[bb5] Darensbourg, D. J. & Frantz, E. B. (2007). *Inorg. Chem.* **46**, 5967–5978. 10.1021/ic7003968 17579473

[bb6] Flack, H. D. (1983). *Acta Cryst.* A**39**, 876–881.

[bb7] Habibi, M. H., Askari, E., Chantrapromma, S. & Fun, H.-K. (2007). *Acta Cryst.* E**63**, m2905–m2906.

[bb8] Kawamoto, T., Nishiwaki, M., Tsunekawa, Y., Nozaki, K. & Konno, T. (2008). *Inorg. Chem.* **47**, 3095–3104. 10.1021/ic7020758 18345616

[bb9] Lipscomb, W. N. & Sträter, N. (1996). *Chem. Rev.* **96**, 2375–2434. 10.1021/cr950042j 11848831

[bb10] Qiu, X.-Y. (2006*a*). *Acta Cryst.* E**62**, m717–m718.

[bb11] Qiu, X.-Y. (2006*b*). *Acta Cryst.* E**62**, m2173–m2174.

[bb12] Sheldrick, G. M. (2004). *SADABS* University of Göttingen, Germany.

[bb13] Sheldrick, G. M. (2008). *Acta Cryst.* A**64**, 112–122. 10.1107/S0108767307043930 18156677

[bb14] Tomat, E., Cuesta, L., Lynch, V. M. & Sessler, J. L. (2007). *Inorg. Chem.* **46**, 6224–6226. 10.1021/ic700933p 17630733

[bb15] Wei, Y.-J., Wang, F.-W. & Zhu, Q.-Y. (2007). *Acta Cryst.* E**63**, m654–m655.

[bb16] Wu, J.-C., Liu, S.-X., Keene, T. D., Neels, A., Mereacre, V., Powell, A. K. & Decurtins, S. (2008). *Inorg. Chem.* **47**, 3452–3459. 10.1021/ic800138x 18370379

[bb17] Yuan, M., Zhao, F., Zhang, W., Wang, Z.-M. & Gao, S. (2007). *Inorg. Chem.* **46**, 11235–11242. 10.1021/ic701655w 18031035

[bb18] Zhu, Q.-Y., Wei, Y.-J. & Wang, F.-W. (2007). *Acta Cryst.* E**63**, m1431–m1432.

[bb19] Zhu, X.-W. & Yang, X.-Z. (2008*a*). *Acta Cryst.* E**64**, m1090–m1091. 10.1107/S1600536808023659 PMC2961998 21203068

[bb20] Zhu, X.-W. & Yang, X.-Z. (2008*b*). *Acta Cryst.* E**64**, m1092–m1093. 10.1107/S1600536808023672 PMC2961999 21203069

[bb21] Zhu, X.-W. & Yang, X.-Z. (2008*c*). *Acta Cryst.* E**64**, m1094–m1095. 10.1107/S1600536808023660 PMC2962000 21203070

[bb22] Zhu, X.-W. & Yang, X.-Z. (2008*d*). *Acta Cryst.* E**64**, m1096–m1097. 10.1107/S1600536808023684 PMC2962001 21203071

